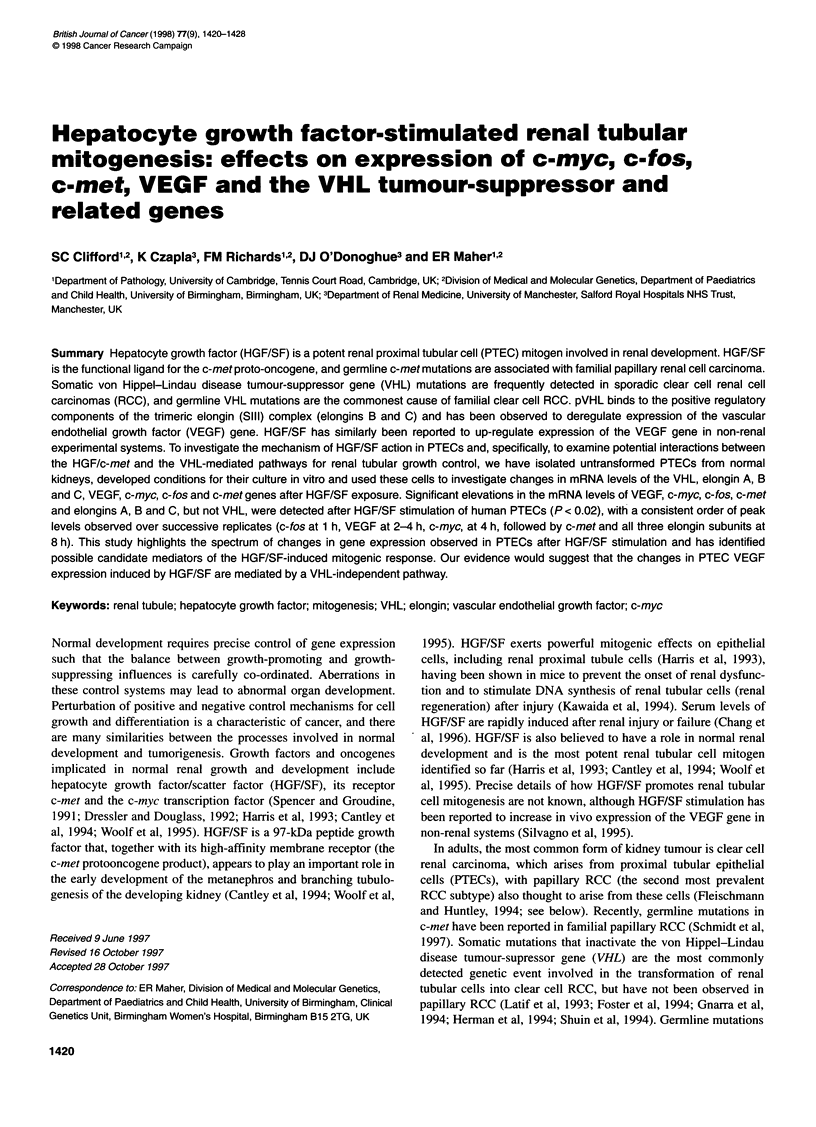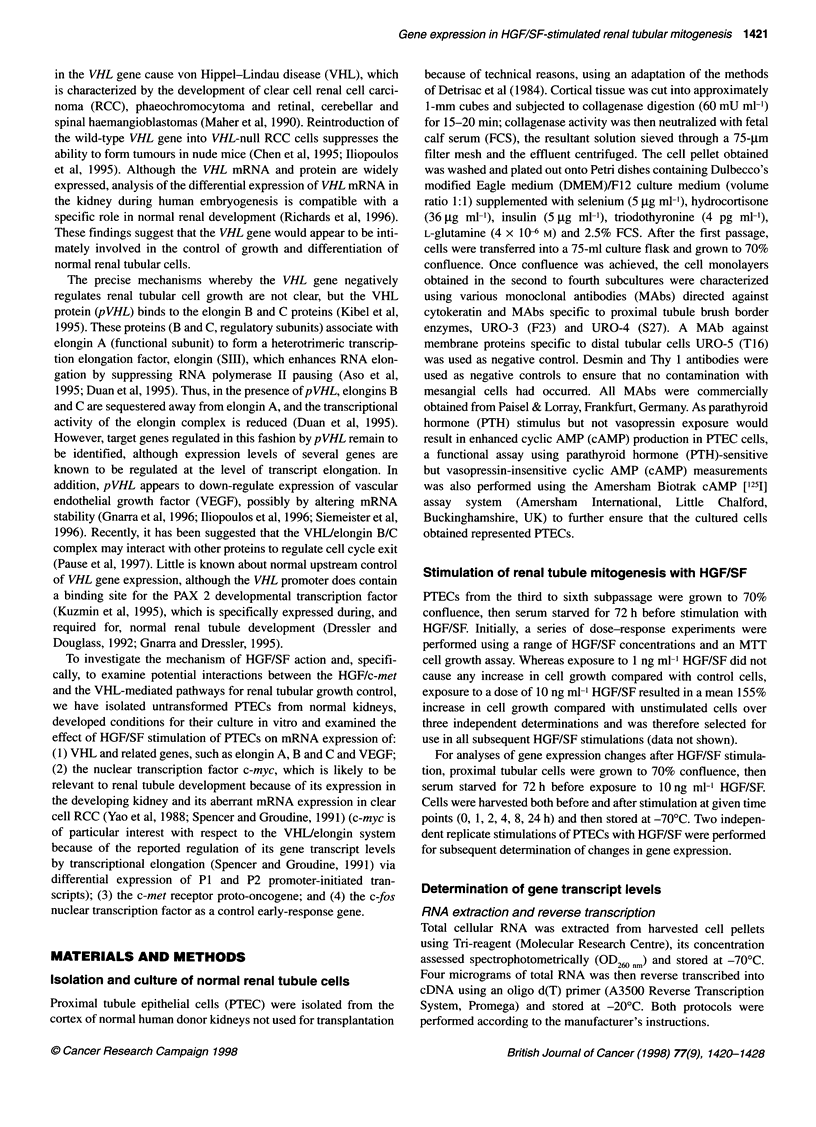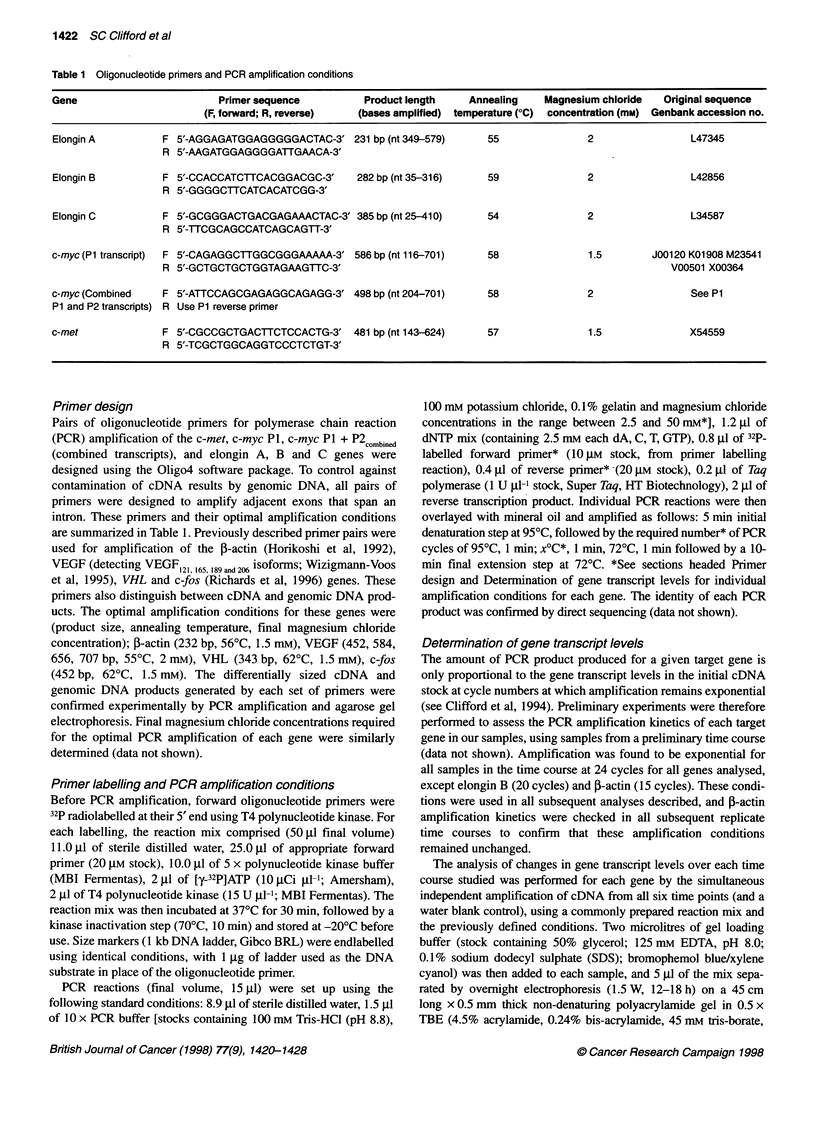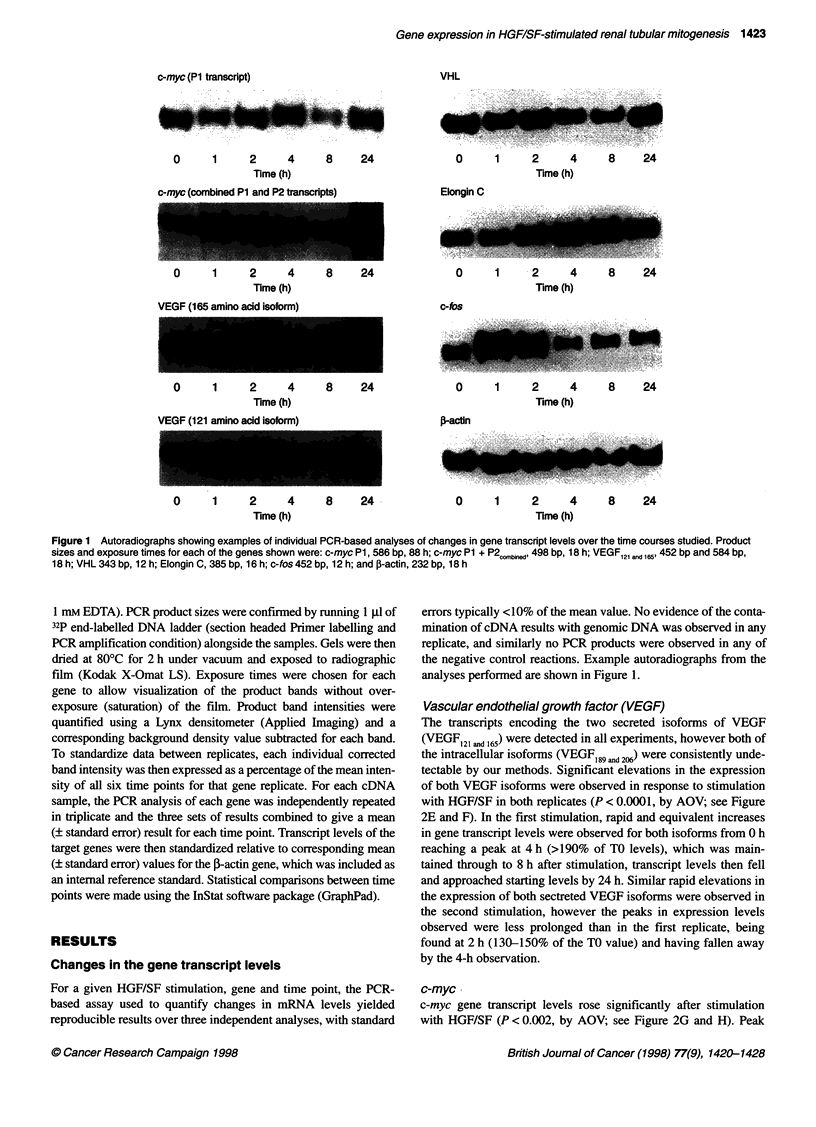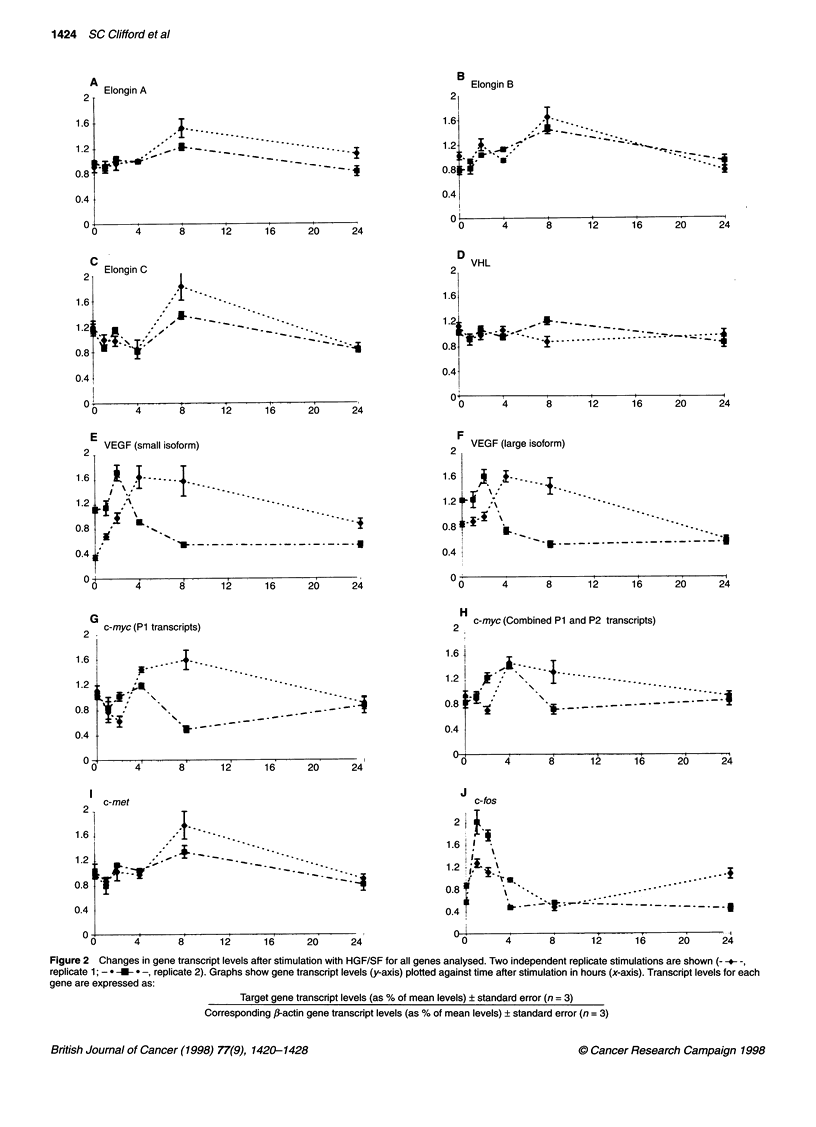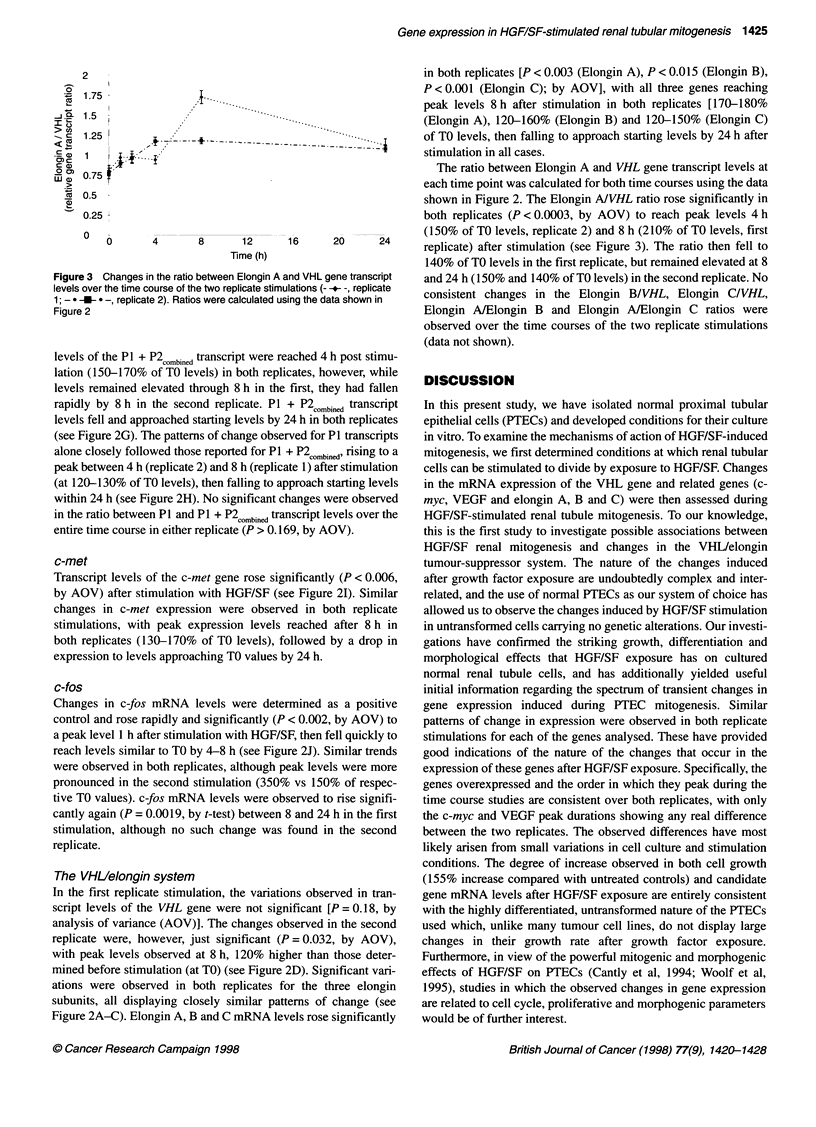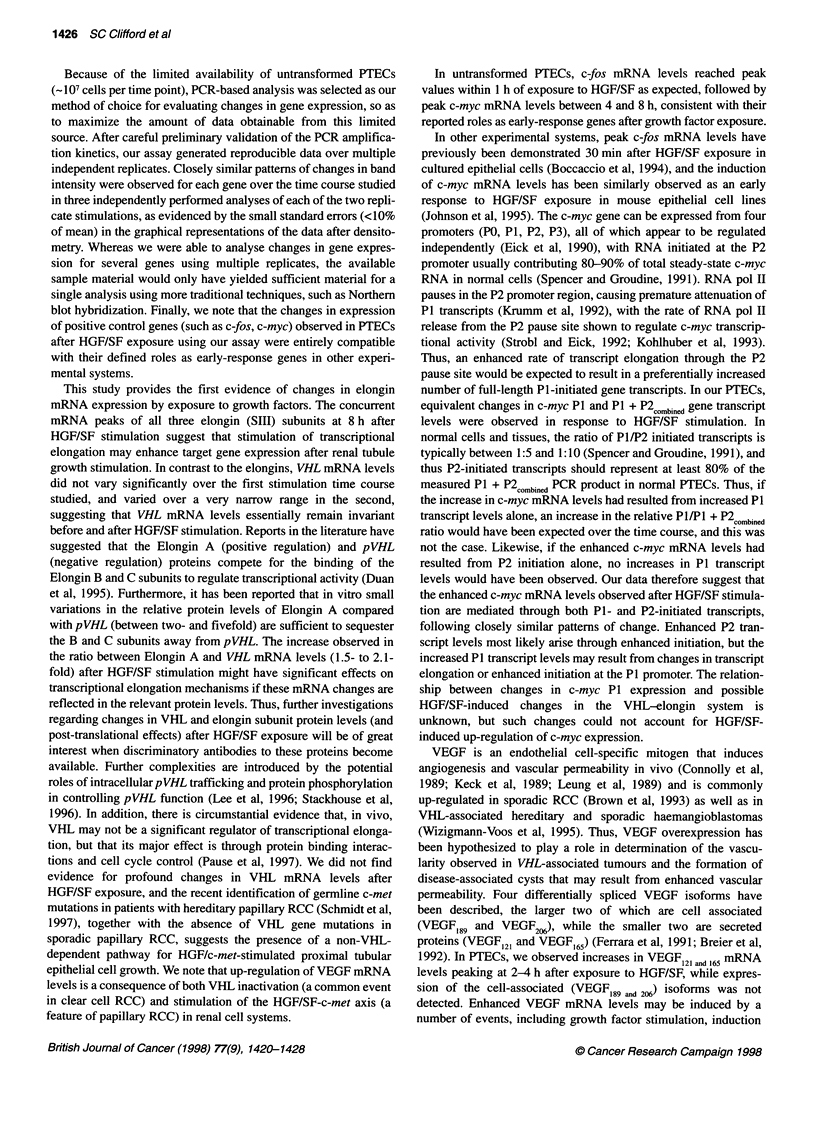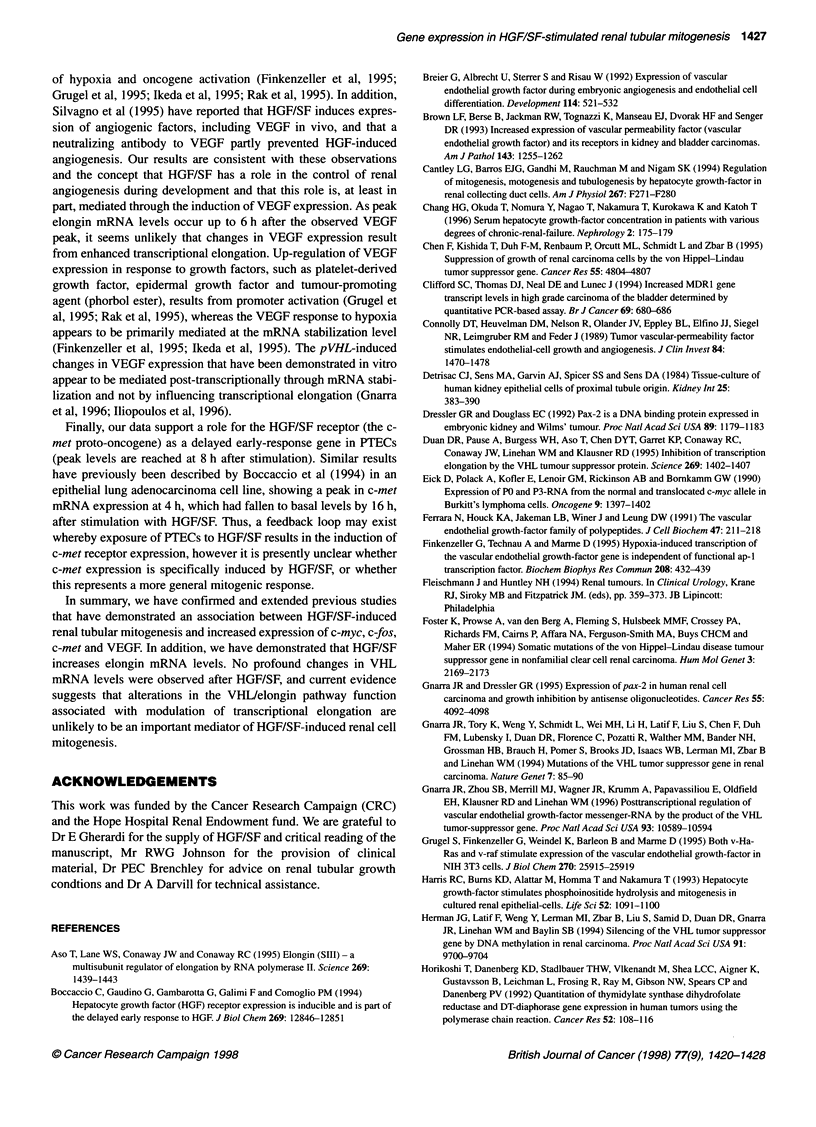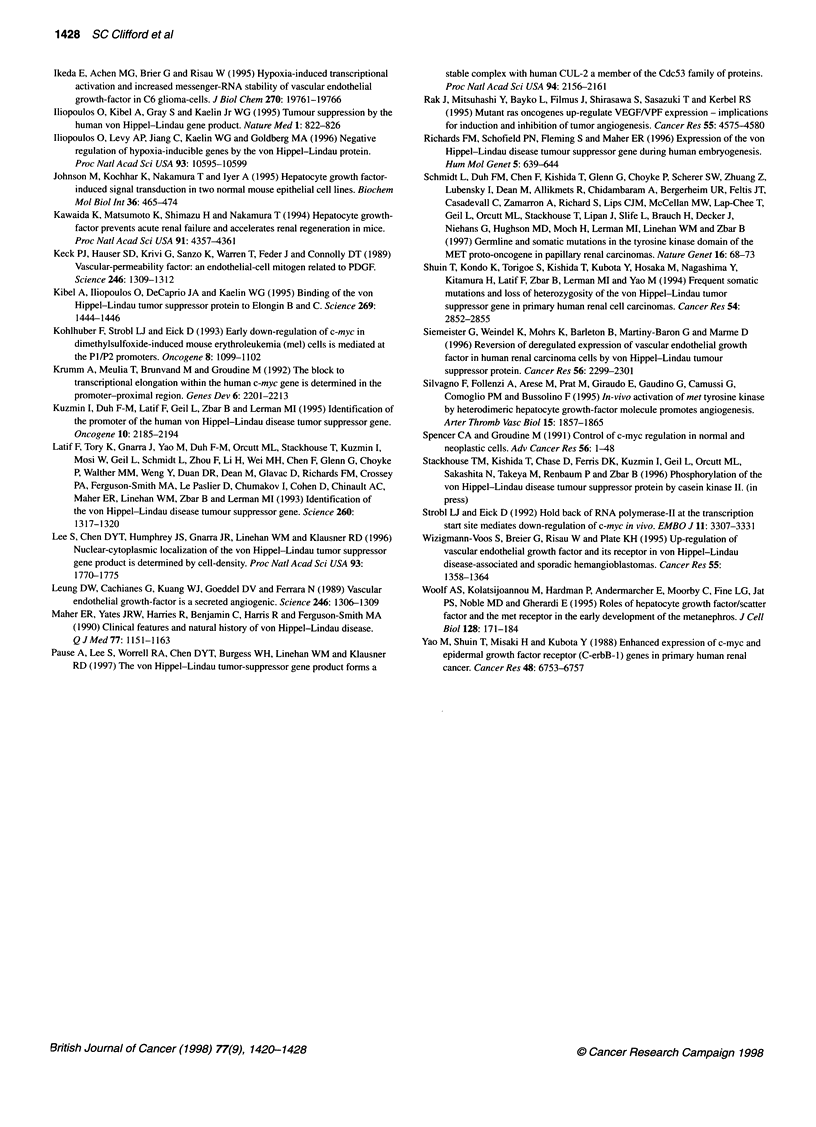# Hepatocyte growth factor-stimulated renal tubular mitogenesis: effects on expression of c-myc, c-fos, c-met, VEGF and the VHL tumour-suppressor and related genes.

**DOI:** 10.1038/bjc.1998.235

**Published:** 1998-05

**Authors:** S. C. Clifford, K. Czapla, F. M. Richards, D. J. O'Donoghue, E. R. Maher

**Affiliations:** Department of Pathology, University of Cambridge, UK.

## Abstract

**Images:**


British Journal of Cancer (1998) 77(9), 1420–1428
© 1998 Cancer Research Campaign

### Hepatocyte growth factor-stimulated renal tubular mitogenesis: effects on expression of c-*myc*, c-*fos*, c-*met*, VEGF and the VHL tumour-suppressor and related genes

**SC Clifford[1,2], K Czapla[3], FM Richards[1,2], DJ O'Donoghue[3] and ER Maher[1,2]**

[1]Department of Pathology, University of Cambridge, Tennis Court Road, Cambridge, UK; [2]Division of Medical and Molecular Genetics, Department of Paediatrics and Child Health, University of Birmingham, Birmingham, UK; [3]Department of Renal Medicine, University of Manchester, Salford Royal Hospitals NHS Trust, Manchester, UK

**Summary** Hepatocyte growth factor (HGF/SF) is a potent renal proximal tubular cell (PTEC) mitogen involved in renal development. HGF/SF is the functional ligand for the c-*met* proto-oncogene, and germline c-*met* mutations are associated with familial papillary renal cell carcinoma. Somatic von Hippel–Lindau disease tumour-suppressor gene (VHL) mutations are frequently detected in sporadic clear cell renal cell carcinomas (RCC), and germline VHL mutations are the commonest cause of familial clear cell RCC. pVHL binds to the positive regulatory components of the trimeric elongin (SIII) complex (elongins B and C) and has been observed to deregulate expression of the vascular endothelial growth factor (VEGF) gene. HGF/SF has similarly been reported to up-regulate expression of the VEGF gene in non-renal experimental systems. To investigate the mechanism of HGF/SF action in PTECs and, specifically, to examine potential interactions between the HGF/c-*met* and the VHL-mediated pathways for renal tubular growth control, we have isolated untransformed PTECs from normal kidneys, developed conditions for their culture in vitro and used these cells to investigate changes in mRNA levels of the VHL, elongin A, B and C, VEGF, c-*myc*, c-*fos* and c-*met* genes after HGF/SF exposure. Significant elevations in the mRNA levels of VEGF, c-*myc*, c-*fos*, c-*met* and elongins A, B and C, but not VHL, were detected after HGF/SF stimulation of human PTECs ($P < 0.02$), with a consistent order of peak levels observed over successive replicates (c-*fos* at 1 h, VEGF at 2–4 h, c-*myc*, at 4 h, followed by c-*met* and all three elongin subunits at 8 h). This study highlights the spectrum of changes in gene expression observed in PTECs after HGF/SF stimulation and has identified possible candidate mediators of the HGF/SF-induced mitogenic response. Our evidence would suggest that the changes in PTEC VEGF expression induced by HGF/SF are mediated by a VHL-independent pathway.

**Keywords:** renal tubule; hepatocyte growth factor; mitogenesis; VHL; elongin; vascular endothelial growth factor; c-*myc*

Normal development requires precise control of gene expression such that the balance between growth-promoting and growth-suppressing influences is carefully co-ordinated. Aberrations in these control systems may lead to abnormal organ development. Perturbation of positive and negative control mechanisms for cell growth and differentiation is a characteristic of cancer, and there are many similarities between the processes involved in normal development and tumorigenesis. Growth factors and oncogenes implicated in normal renal growth and development include hepatocyte growth factor/scatter factor (HGF/SF), its receptor c-*met* and the c-*myc* transcription factor (Spencer and Groudine, 1991; Dressler and Douglass, 1992; Harris et al, 1993; Cantley et al, 1994; Woolf et al, 1995). HGF/SF is a 97-kDa peptide growth factor that, together with its high-affinity membrane receptor (the c-*met* protooncogene product), appears to play an important role in the early development of the metanephros and branching tubulogenesis of the developing kidney (Cantley et al, 1994; Woolf et al, 1995). HGF/SF exerts powerful mitogenic effects on epithelial cells, including renal proximal tubule cells (Harris et al, 1993), having been shown in mice to prevent the onset of renal dysfunction and to stimulate DNA synthesis of renal tubular cells (renal regeneration) after injury (Kawaida et al, 1994). Serum levels of HGF/SF are rapidly induced after renal injury or failure (Chang et al, 1996). HGF/SF is also believed to have a role in normal renal development and is the most potent renal tubular cell mitogen identified so far (Harris et al, 1993; Cantley et al, 1994; Woolf et al, 1995). Precise details of how HGF/SF promotes renal tubular cell mitogenesis are not known, although HGF/SF stimulation has been reported to increase in vivo expression of the VEGF gene in non-renal systems (Silvagno et al, 1995).

In adults, the most common form of kidney tumour is clear cell renal carcinoma, which arises from proximal tubular epithelial cells (PTECs), with papillary RCC (the second most prevalent RCC subtype) also thought to arise from these cells (Fleischmann and Huntley, 1994; see below). Recently, germline mutations in c-*met* have been reported in familial papillary RCC (Schmidt et al, 1997). Somatic mutations that inactivate the von Hippel–Lindau disease tumour-supressor gene (*VHL*) are the most commonly detected genetic event involved in the transformation of renal tubular cells into clear cell RCC, but have not been observed in papillary RCC (Latif et al, 1993; Foster et al, 1994; Gnarra et al, 1994; Herman et al, 1994; Shuin et al, 1994). Germline mutations

*Received 9 June 1997*
*Revised 16 October 1997*
*Accepted 28 October 1997*

*Correspondence to:* ER Maher, Division of Medical and Molecular Genetics, Department of Paediatrics and Child Health, University of Birmingham, Clinical Genetics Unit, Birmingham Women's Hospital, Birmingham B15 2TG, UK

in the *VHL* gene cause von Hippel–Lindau disease (VHL), which is characterized by the development of clear cell renal cell carcinoma (RCC), phaeochromocytoma and retinal, cerebellar and spinal haemangioblastomas (Maher et al, 1990). Reintroduction of the wild-type *VHL* gene into *VHL*-null RCC cells suppresses the ability to form tumours in nude mice (Chen et al, 1995; Iliopoulos et al, 1995). Although the *VHL* mRNA and protein are widely expressed, analysis of the differential expression of *VHL* mRNA in the kidney during human embryogenesis is compatible with a specific role in normal renal development (Richards et al, 1996). These findings suggest that the *VHL* gene would appear to be intimately involved in the control of growth and differentiation of normal renal tubular cells.

The precise mechanisms whereby the *VHL* gene negatively regulates renal tubular cell growth are not clear, but the VHL protein (*pVHL*) binds to the elongin B and C proteins (Kibel et al, 1995). These proteins (B and C, regulatory subunits) associate with elongin A (functional subunit) to form a heterotrimeric transcription elongation factor, elongin (SIII), which enhances RNA elongation by suppressing RNA polymerase II pausing (Aso et al, 1995; Duan et al, 1995). Thus, in the presence of *pVHL*, elongins B and C are sequestered away from elongin A, and the transcriptional activity of the elongin complex is reduced (Duan et al, 1995). However, target genes regulated in this fashion by *pVHL* remain to be identified, although expression levels of several genes are known to be regulated at the level of transcript elongation. In addition, *pVHL* appears to down-regulate expression of vascular endothelial growth factor (VEGF), possibly by altering mRNA stability (Gnarra et al, 1996; Iliopoulos et al, 1996; Siemeister et al, 1996). Recently, it has been suggested that the VHL/elongin B/C complex may interact with other proteins to regulate cell cycle exit (Pause et al, 1997). Little is known about normal upstream control of *VHL* gene expression, although the *VHL* promoter does contain a binding site for the PAX 2 developmental transcription factor (Kuzmin et al, 1995), which is specifically expressed during, and required for, normal renal tubule development (Dressler and Douglass, 1992; Gnarra and Dressler, 1995).

To investigate the mechanism of HGF/SF action and, specifically, to examine potential interactions between the HGF/*c-met* and the VHL-mediated pathways for renal tubular growth control, we have isolated untransformed PTECs from normal kidneys, developed conditions for their culture in vitro and examined the effect of HGF/SF stimulation of PTECs on mRNA expression of: (1) VHL and related genes, such as elongin A, B and C and VEGF; (2) the nuclear transcription factor c-*myc*, which is likely to be relevant to renal tubule development because of its expression in the developing kidney and its aberrant mRNA expression in clear cell RCC (Yao et al, 1988; Spencer and Groudine, 1991) (c-*myc* is of particular interest with respect to the VHL/elongin system because of the reported regulation of its gene transcript levels by transcriptional elongation (Spencer and Groudine, 1991) via differential expression of P1 and P2 promoter-initiated transcripts); (3) the c-*met* receptor proto-oncogene; and (4) the c-*fos* nuclear transcription factor as a control early-response gene.

### MATERIALS AND METHODS

### Isolation and culture of normal renal tubule cells

Proximal tubule epithelial cells (PTEC) were isolated from the cortex of normal human donor kidneys not used for transplantation because of technical reasons, using an adaptation of the methods of Detrisac et al (1984). Cortical tissue was cut into approximately 1-mm cubes and subjected to collagenase digestion (60 mU $ml^{-1}$) for 15–20 min; collagenase activity was then neutralized with fetal calf serum (FCS), the resultant solution sieved through a 75-μm filter mesh and the effluent centrifuged. The cell pellet obtained was washed and plated out onto Petri dishes containing Dulbecco's modified Eagle medium (DMEM)/F12 culture medium (volume ratio 1:1) supplemented with selenium (5 μg $ml^{-1}$), hydrocortisone (36 μg $ml^{-1}$), insulin (5 μg $ml^{-1}$), triodothyronine (4 pg $ml^{-1}$), L-glutamine ($4 \times 10^{-6}$ M) and 2.5% FCS. After the first passage, cells were transferred into a 75-ml culture flask and grown to 70% confluence. Once confluence was achieved, the cell monolayers obtained in the second to fourth subcultures were characterized using various monoclonal antibodies (MAbs) directed against cytokeratin and MAbs specific to proximal tubule brush border enzymes, URO-3 (F23) and URO-4 (S27). A MAb against membrane proteins specific to distal tubular cells URO-5 (T16) was used as negative control. Desmin and Thy 1 antibodies were used as negative controls to ensure that no contamination with mesangial cells had occurred. All MAbs were commercially obtained from Paisel & Lorray, Frankfurt, Germany. As parathyroid hormone (PTH) stimulus but not vasopressin exposure would result in enhanced cyclic AMP (cAMP) production in PTEC cells, a functional assay using parathyroid hormone (PTH)-sensitive but vasopressin-insensitive cyclic AMP (cAMP) measurements was also performed using the Amersham Biotrak cAMP [$^{125}$I] assay system (Amersham International, Little Chalford, Buckinghamshire, UK) to further ensure that the cultured cells obtained represented PTECs.

### Stimulation of renal tubule mitogenesis with HGF/SF

PTECs from the third to sixth subpassage were grown to 70% confluence, then serum starved for 72 h before stimulation with HGF/SF. Initially, a series of dose–response experiments were performed using a range of HGF/SF concentrations and an MTT cell growth assay. Whereas exposure to 1 ng $ml^{-1}$ HGF/SF did not cause any increase in cell growth compared with control cells, exposure to a dose of 10 ng $ml^{-1}$ HGF/SF resulted in a mean 155% increase in cell growth compared with unstimulated cells over three independent determinations and was therefore selected for use in all subsequent HGF/SF stimulations (data not shown).

For analyses of gene expression changes after HGF/SF stimulation, proximal tubular cells were grown to 70% confluence, then serum starved for 72 h before exposure to 10 ng $ml^{-1}$ HGF/SF. Cells were harvested both before and after stimulation at given time points (0, 1, 2, 4, 8, 24 h) and then stored at –70°C. Two independent replicate stimulations of PTECs with HGF/SF were performed for subsequent determination of changes in gene expression.

### Determination of gene transcript levels

#### *RNA extraction and reverse transcription*

Total cellular RNA was extracted from harvested cell pellets using Tri-reagent (Molecular Research Centre), its concentration assessed spectrophotometrically ($OD_{260\ nm}$) and stored at –70°C. Four micrograms of total RNA was then reverse transcribed into cDNA using an oligo d(T) primer (A3500 Reverse Transcription System, Promega) and stored at –20°C. Both protocols were performed according to the manufacturer's instructions.

© Cancer Research Campaign 1998

**Table 1** Oligonucleotide primers and PCR amplification conditions

| Gene | Primer sequence (F, forward; R, reverse) | Product length (bases amplified) | Annealing temperature (°C) | Magnesium chloride concentration (mM) | Original sequence Genbank accession no. |
|---|---|---|---|---|---|
| Elongin A | F 5′-AGGAGATGGAGGGGGACTAC-3′<br>R 5′-AAGATGGAGGGGATTGAACA-3′ | 231 bp (nt 349–579) | 55 | 2 | L47345 |
| Elongin B | F 5′-CCACCATCTTCACGGACGC-3′<br>R 5′-GGGGCTTCATCACATCGG-3′ | 282 bp (nt 35–316) | 59 | 2 | L42856 |
| Elongin C | F 5′-GCGGGACTGACGAGAAACTAC-3′<br>R 5′-TTCGCAGCCATCAGCAGTT-3′ | 385 bp (nt 25–410) | 54 | 2 | L34587 |
| c-*myc* (P1 transcript) | F 5′-CAGAGGCTTGGCGGGAAAAA-3′<br>R 5′-GCTGCTGCTGGTAGAAGTTC-3′ | 586 bp (nt 116–701) | 58 | 1.5 | J00120 K01908 M23541 V00501 X00364 |
| c-*myc* (Combined P1 and P2 transcripts) | F 5′-ATTCCAGCGAGAGGCAGAGG-3′<br>R Use P1 reverse primer | 498 bp (nt 204–701) | 58 | 2 | See P1 |
| c-*met* | F 5′-CGCCGCTGACTTCTCCACTG-3′<br>R 5′-TCGCTGGCAGGTCCCTCTGT-3′ | 481 bp (nt 143–624) | 57 | 1.5 | X54559 |

### Primer design

Pairs of oligonucleotide primers for polymerase chain reaction (PCR) amplification of the c-*met*, c-*myc* P1, c-*myc* P1 + $P2_{combined}$ (combined transcripts), and elongin A, B and C genes were designed using the Oligo4 software package. To control against contamination of cDNA results by genomic DNA, all pairs of primers were designed to amplify adjacent exons that span an intron. These primers and their optimal amplification conditions are summarized in Table 1. Previously described primer pairs were used for amplification of the β-actin (Horikoshi et al, 1992), VEGF (detecting $VEGF_{121, 165, 189 \text{ and } 206}$ isoforms; Wizigmann-Voos et al, 1995), *VHL* and c-*fos* (Richards et al, 1996) genes. These primers also distinguish between cDNA and genomic DNA products. The optimal amplification conditions for these genes were (product size, annealing temperature, final magnesium chloride concentration); β-actin (232 bp, 56°C, 1.5 mM), VEGF (452, 584, 656, 707 bp, 55°C, 2 mM), VHL (343 bp, 62°C, 1.5 mM), c-*fos* (452 bp, 62°C, 1.5 mM). The differentially sized cDNA and genomic DNA products generated by each set of primers were confirmed experimentally by PCR amplification and agarose gel electrophoresis. Final magnesium chloride concentrations required for the optimal PCR amplification of each gene were similarly determined (data not shown).

### Primer labelling and PCR amplification conditions

Before PCR amplification, forward oligonucleotide primers were $^{32}P$ radiolabelled at their 5′ end using T4 polynucleotide kinase. For each labelling, the reaction mix comprised (50 μl final volume) 11.0 μl of sterile distilled water, 25.0 μl of appropriate forward primer (20 μM stock), 10.0 μl of 5 × polynucleotide kinase buffer (MBI Fermentas), 2 μl of [γ-$^{32}P$]ATP (10 μCi $\mu l^{-1}$; Amersham), 2 μl of T4 polynucleotide kinase (15 U $\mu l^{-1}$; MBI Fermentas). The reaction mix was then incubated at 37°C for 30 min, followed by a kinase inactivation step (70°C, 10 min) and stored at –20°C before use. Size markers (1 kb DNA ladder, Gibco BRL) were endlabelled using identical conditions, with 1 μg of ladder used as the DNA substrate in place of the oligonucleotide primer.

PCR reactions (final volume, 15 μl) were set up using the following standard conditions: 8.9 μl of sterile distilled water, 1.5 μl of 10 × PCR buffer [stocks containing 100 mM Tris-HCl (pH 8.8), 100 mM potassium chloride, 0.1% gelatin and magnesium chloride concentrations in the range between 2.5 and 50 mM*], 1.2 μl of dNTP mix (containing 2.5 mM each dA, C, T, GTP), 0.8 μl of $^{32}P$-labelled forward primer* (10 μM stock, from primer labelling reaction), 0.4 μl of reverse primer* (20 μM stock), 0.2 μl of *Taq* polymerase (1 U $\mu l^{-1}$ stock, Super *Taq*, HT Biotechnology), 2 μl of reverse transcription product. Individual PCR reactions were then overlayed with mineral oil and amplified as follows: 5 min initial denaturation step at 95°C, followed by the required number* of PCR cycles of 95°C, 1 min; *x*°C*, 1 min, 72°C, 1 min followed by a 10-min final extension step at 72°C. *See sections headed Primer design and Determination of gene transcript levels for individual amplification conditions for each gene. The identity of each PCR product was confirmed by direct sequencing (data not shown).

### Determination of gene transcript levels

The amount of PCR product produced for a given target gene is only proportional to the gene transcript levels in the initial cDNA stock at cycle numbers at which amplification remains exponential (see Clifford et al, 1994). Preliminary experiments were therefore performed to assess the PCR amplification kinetics of each target gene in our samples, using samples from a preliminary time course (data not shown). Amplification was found to be exponential for all samples in the time course at 24 cycles for all genes analysed, except elongin B (20 cycles) and β-actin (15 cycles). These conditions were used in all subsequent analyses described, and β-actin amplification kinetics were checked in all subsequent replicate time courses to confirm that these amplification conditions remained unchanged.

The analysis of changes in gene transcript levels over each time course studied was performed for each gene by the simultaneous independent amplification of cDNA from all six time points (and a water blank control), using a commonly prepared reaction mix and the previously defined conditions. Two microlitres of gel loading buffer (stock containing 50% glycerol; 125 mM EDTA, pH 8.0; 0.1% sodium dodecyl sulphate (SDS); bromophemol blue/xylene cyanol) was then added to each sample, and 5 μl of the mix separated by overnight electrophoresis (1.5 W, 12–18 h) on a 45 cm long × 0.5 mm thick non-denaturing polyacrylamide gel in 0.5 × TBE (4.5% acrylamide, 0.24% bis-acrylamide, 45 mM tris-borate,

 © Cancer Research Campaign 1998

**Figure 1** Autoradiographs showing examples of individual PCR-based analyses of changes in gene transcript levels over the time courses studied. Product sizes and exposure times for each of the genes shown were: *c-myc* P1, 586 bp, 88 h; *c-myc* P1 + $P2_{combined}$, 498 bp, 18 h; $VEGF_{121 \text{ and } 165}$, 452 bp and 584 bp, 18 h; VHL 343 bp, 12 h; Elongin C, 385 bp, 16 h; *c-fos* 452 bp, 12 h; and β-actin, 232 bp, 18 h

1 mM EDTA). PCR product sizes were confirmed by running 1 µl of $^{32}P$ end-labelled DNA ladder (section headed Primer labelling and PCR amplification condition) alongside the samples. Gels were then dried at 80°C for 2 h under vacuum and exposed to radiographic film (Kodak X-Omat LS). Exposure times were chosen for each gene to allow visualization of the product bands without over-exposure (saturation) of the film. Product band intensities were quantified using a Lynx densitometer (Applied Imaging) and a corresponding background density value subtracted for each band. To standardize data between replicates, each individual corrected band intensity was then expressed as a percentage of the mean intensity of all six time points for that gene replicate. For each cDNA sample, the PCR analysis of each gene was independently repeated in triplicate and the three sets of results combined to give a mean (± standard error) result for each time point. Transcript levels of the target genes were then standardized relative to corresponding mean (± standard error) values for the β-actin gene, which was included as an internal reference standard. Statistical comparisons between time points were made using the InStat software package (GraphPad).

### RESULTS

### Changes in the gene transcript levels

For a given HGF/SF stimulation, gene and time point, the PCR-based assay used to quantify changes in mRNA levels yielded reproducible results over three independent analyses, with standard errors typically <10% of the mean value. No evidence of the contamination of cDNA results with genomic DNA was observed in any replicate, and similarly no PCR products were observed in any of the negative control reactions. Example autoradiographs from the analyses performed are shown in Figure 1.

#### *Vascular endothelial growth factor (VEGF)*

The transcripts encoding the two secreted isoforms of VEGF ($VEGF_{121 \text{ and } 165}$) were detected in all experiments, however both of the intracellular isoforms ($VEGF_{189 \text{ and } 206}$) were consistently undetectable by our methods. Significant elevations in the expression of both VEGF isoforms were observed in response to stimulation with HGF/SF in both replicates ($P < 0.0001$, by AOV; see Figure 2E and F). In the first stimulation, rapid and equivalent increases in gene transcript levels were observed for both isoforms from 0 h reaching a peak at 4 h (>190% of T0 levels), which was maintained through to 8 h after stimulation, transcript levels then fell and approached starting levels by 24 h. Similar rapid elevations in the expression of both sectreted VEGF isoforms were observed in the second stimulation, however the peaks in expression levels observed were less prolonged than in the first replicate, being found at 2 h (130–150% of the T0 value) and having fallen away by the 4-h observation.

#### *c-myc*

*c-myc* gene transcript levels rose significantly after stimulation with HGF/SF ($P < 0.002$, by AOV; see Figure 2G and H). Peak

© Cancer Research Campaign 1998 

**Figure 2** Changes in gene transcript levels after stimulation with HGF/SF for all genes analysed. Two independent replicate stimulations are shown (- -◆- -, replicate 1; – • -■- • –, replicate 2). Graphs show gene transcript levels (*y*-axis) plotted against time after stimulation in hours (*x*-axis). Transcript levels for each gene are expressed as:

$$\frac{\text{Target gene transcript levels (as \% of mean levels)} \pm \text{standard error } (n = 3)}{\text{Corresponding } \beta\text{-actin gene transcript levels (as \% of mean levels)} \pm \text{standard error } (n = 3)}$$

 © Cancer Research Campaign 1998

**Figure 3** Changes in the ratio between Elongin A and VHL gene transcript levels over the time course of the two replicate stimulations (- -◆- -, replicate 1; – • -■- • –, replicate 2). Ratios were calculated using the data shown in Figure 2

levels of the P1 + $P2_{combined}$ transcript were reached 4 h post stimulation (150–170% of T0 levels) in both replicates, however, while levels remained elevated through 8 h in the first, they had fallen rapidly by 8 h in the second replicate. P1 + $P2_{combined}$ transcript levels fell and approached starting levels by 24 h in both replicates (see Figure 2G). The patterns of change observed for P1 transcripts alone closely followed those reported for P1 + $P2_{combined}$, rising to a peak between 4 h (replicate 2) and 8 h (replicate 1) after stimulation (at 120–130% of T0 levels), then falling to approach starting levels within 24 h (see Figure 2H). No significant changes were observed in the ratio between P1 and P1 + $P2_{combined}$ transcript levels over the entire time course in either replicate ($P > 0.169$, by AOV).

### c-met

Transcript levels of the c-*met* gene rose significantly ($P < 0.006$, by AOV) after stimulation with HGF/SF (see Figure 2I). Similar changes in c-*met* expression were observed in both replicate stimulations, with peak expression levels reached after 8 h in both replicates (130–170% of T0 levels), followed by a drop in expression to levels approaching T0 values by 24 h.

### c-fos

Changes in c-*fos* mRNA levels were determined as a positive control and rose rapidly and significantly ($P < 0.002$, by AOV) to a peak level 1 h after stimulation with HGF/SF, then fell quickly to reach levels similar to T0 by 4–8 h (see Figure 2J). Similar trends were observed in both replicates, although peak levels were more pronounced in the second stimulation (350% vs 150% of respective T0 values). c-*fos* mRNA levels were observed to rise significantly again ($P = 0.0019$, by *t*-test) between 8 and 24 h in the first stimulation, although no such change was found in the second replicate.

### The VHL/elongin system

In the first replicate stimulation, the variations observed in transcript levels of the *VHL* gene were not significant [$P = 0.18$, by analysis of variance (AOV)]. The changes observed in the second replicate were, however, just significant ($P = 0.032$, by AOV), with peak levels observed at 8 h, 120% higher than those determined before stimulation (at T0) (see Figure 2D). Significant variations were observed in both replicates for the three elongin subunits, all displaying closely similar patterns of change (see Figure 2A–C). Elongin A, B and C mRNA levels rose significantly in both replicates [$P < 0.003$ (Elongin A), $P < 0.015$ (Elongin B), $P < 0.001$ (Elongin C); by AOV], with all three genes reaching peak levels 8 h after stimulation in both replicates [170–180% (Elongin A), 120–160% (Elongin B) and 120–150% (Elongin C) of T0 levels, then falling to approach starting levels by 24 h after stimulation in all cases.

The ratio between Elongin A and *VHL* gene transcript levels at each time point was calculated for both time courses using the data shown in Figure 2. The Elongin A/*VHL* ratio rose significantly in both replicates ($P < 0.0003$, by AOV) to reach peak levels 4 h (150% of T0 levels, replicate 2) and 8 h (210% of T0 levels, first replicate) after stimulation (see Figure 3). The ratio then fell to 140% of T0 levels in the first replicate, but remained elevated at 8 and 24 h (150% and 140% of T0 levels) in the second replicate. No consistent changes in the Elongin B/*VHL*, Elongin C/*VHL*, Elongin A/Elongin B and Elongin A/Elongin C ratios were observed over the time courses of the two replicate stimulations (data not shown).

### DISCUSSION

In this present study, we have isolated normal proximal tubular epithelial cells (PTECs) and developed conditions for their culture in vitro. To examine the mechanisms of action of HGF/SF-induced mitogenesis, we first determined conditions at which renal tubular cells can be stimulated to divide by exposure to HGF/SF. Changes in the mRNA expression of the VHL gene and related genes (c-*myc*, VEGF and elongin A, B and C) were then assessed during HGF/SF-stimulated renal tubule mitogenesis. To our knowledge, this is the first study to investigate possible associations between HGF/SF renal mitogenesis and changes in the VHL/elongin tumour-suppressor system. The nature of the changes induced after growth factor exposure are undoubtedly complex and interrelated, and the use of normal PTECs as our system of choice has allowed us to observe the changes induced by HGF/SF stimulation in untransformed cells carrying no genetic alterations. Our investigations have confirmed the striking growth, differentiation and morphological effects that HGF/SF exposure has on cultured normal renal tubule cells, and has additionally yielded useful initial information regarding the spectrum of transient changes in gene expression induced during PTEC mitogenesis. Similar patterns of change in expression were observed in both replicate stimulations for each of the genes analysed. These have provided good indications of the nature of the changes that occur in the expression of these genes after HGF/SF exposure. Specifically, the genes overexpressed and the order in which they peak during the time course studies are consistent over both replicates, with only the c-*myc* and VEGF peak durations showing any real difference between the two replicates. The observed differences have most likely arisen from small variations in cell culture and stimulation conditions. The degree of increase observed in both cell growth (155% increase compared with untreated controls) and candidate gene mRNA levels after HGF/SF exposure are entirely consistent with the highly differentiated, untransformed nature of the PTECs used which, unlike many tumour cell lines, do not display large changes in their growth rate after growth factor exposure. Furthermore, in view of the powerful mitogenic and morphogenic effects of HGF/SF on PTECs (Cantly et al, 1994; Woolf et al, 1995), studies in which the observed changes in gene expression are related to cell cycle, proliferative and morphogenic parameters would be of further interest.

© Cancer Research Campaign 1998 

Because of the limited availability of untransformed PTECs (~$10^7$ cells per time point), PCR-based analysis was selected as our method of choice for evaluating changes in gene expression, so as to maximize the amount of data obtainable from this limited source. After careful preliminary validation of the PCR amplification kinetics, our assay generated reproducible data over multiple independent replicates. Closely similar patterns of changes in band intensity were observed for each gene over the time course studied in three independently performed analyses of each of the two replicate stimulations, as evidenced by the small standard errors (<10% of mean) in the graphical representations of the data after densitometry. Whereas we were able to analyse changes in gene expression for several genes using multiple replicates, the available sample material would only have yielded sufficient material for a single analysis using more traditional techniques, such as Northern blot hybridization. Finally, we note that the changes in expression of positive control genes (such as c-*fos*, c-*myc*) observed in PTECs after HGF/SF exposure using our assay were entirely compatible with their defined roles as early-response genes in other experimental systems.

This study provides the first evidence of changes in elongin mRNA expression by exposure to growth factors. The concurrent mRNA peaks of all three elongin (SIII) subunits at 8 h after HGF/SF stimulation suggest that stimulation of transcriptional elongation may enhance target gene expression after renal tubule growth stimulation. In contrast to the elongins, *VHL* mRNA levels did not vary significantly over the first stimulation time course studied, and varied over a very narrow range in the second, suggesting that *VHL* mRNA levels essentially remain invariant before and after HGF/SF stimulation. Reports in the literature have suggested that the Elongin A (positive regulation) and *pVHL* (negative regulation) proteins compete for the binding of the Elongin B and C subunits to regulate transcriptional activity (Duan et al, 1995). Furthermore, it has been reported that in vitro small variations in the relative protein levels of Elongin A compared with *pVHL* (between two- and fivefold) are sufficient to sequester the B and C subunits away from *pVHL*. The increase observed in the ratio between Elongin A and *VHL* mRNA levels (1.5- to 2.1-fold) after HGF/SF stimulation might have significant effects on transcriptional elongation mechanisms if these mRNA changes are reflected in the relevant protein levels. Thus, further investigations regarding changes in VHL and elongin subunit protein levels (and post-translational effects) after HGF/SF exposure will be of great interest when discriminatory antibodies to these proteins become available. Further complexities are introduced by the potential roles of intracellular *pVHL* trafficking and protein phosphorylation in controlling *pVHL* function (Lee et al, 1996; Stackhouse et al, 1996). In addition, there is circumstantial evidence that, in vivo, VHL may not be a significant regulator of transcriptional elongation, but that its major effect is through protein binding interactions and cell cycle control (Pause et al, 1997). We did not find evidence for profound changes in VHL mRNA levels after HGF/SF exposure, and the recent identification of germline c-*met* mutations in patients with hereditary papillary RCC (Schmidt et al, 1997), together with the absence of VHL gene mutations in sporadic papillary RCC, suggests the presence of a non-VHL-dependent pathway for HGF/c-*met*-stimulated proximal tubular epithelial cell growth. We note that up-regulation of VEGF mRNA levels is a consequence of both VHL inactivation (a common event in clear cell RCC) and stimulation of the HGF/SF-c-*met* axis (a feature of papillary RCC) in renal cell systems.

In untransformed PTECs, c-*fos* mRNA levels reached peak values within 1 h of exposure to HGF/SF as expected, followed by peak c-*myc* mRNA levels between 4 and 8 h, consistent with their reported roles as early-response genes after growth factor exposure.

In other experimental systems, peak c-*fos* mRNA levels have previously been demonstrated 30 min after HGF/SF exposure in cultured epithelial cells (Boccaccio et al, 1994), and the induction of c-*myc* mRNA levels has been similarly observed as an early response to HGF/SF exposure in mouse epithelial cell lines (Johnson et al, 1995). The c-*myc* gene can be expressed from four promoters (P0, P1, P2, P3), all of which appear to be regulated independently (Eick et al, 1990), with RNA initiated at the P2 promoter usually contributing 80–90% of total steady-state c-*myc* RNA in normal cells (Spencer and Groudine, 1991). RNA pol II pauses in the P2 promoter region, causing premature attenuation of P1 transcripts (Krumm et al, 1992), with the rate of RNA pol II release from the P2 pause site shown to regulate c-*myc* transcriptional activity (Strobl and Eick, 1992; Kohlhuber et al, 1993). Thus, an enhanced rate of transcript elongation through the P2 pause site would be expected to result in a preferentially increased number of full-length P1-initiated gene transcripts. In our PTECs, equivalent changes in c-*myc* P1 and P1 + $P2_{combined}$ gene transcript levels were observed in response to HGF/SF stimulation. In normal cells and tissues, the ratio of P1/P2 initiated transcripts is typically between 1:5 and 1:10 (Spencer and Groudine, 1991), and thus P2-initiated transcripts should represent at least 80% of the measured P1 + $P2_{combined}$ PCR product in normal PTECs. Thus, if the increase in c-*myc* mRNA levels had resulted from increased P1 transcript levels alone, an increase in the relative P1/P1 + $P2_{combined}$ ratio would have been expected over the time course, and this was not the case. Likewise, if the enhanced c-*myc* mRNA levels had resulted from P2 initiation alone, no increases in P1 transcript levels would have been observed. Our data therefore suggest that the enhanced c-*myc* mRNA levels observed after HGF/SF stimulation are mediated through both P1- and P2-initiated transcripts, following closely similar patterns of change. Enhanced P2 transcript levels most likely arise through enhanced initiation, but the increased P1 transcript levels may result from changes in transcript elongation or enhanced initiation at the P1 promoter. The relationship between changes in c-*myc* P1 expression and possible HGF/SF-induced changes in the VHL–elongin system is unknown, but such changes could not account for HGF/SF-induced up-regulation of c-*myc* expression.

VEGF is an endothelial cell-specific mitogen that induces angiogenesis and vascular permeability in vivo (Connolly et al, 1989; Keck et al, 1989; Leung et al, 1989) and is commonly up-regulated in sporadic RCC (Brown et al, 1993) as well as in VHL-associated hereditary and sporadic haemangioblastomas (Wizigmann-Voos et al, 1995). Thus, VEGF overexpression has been hypothesized to play a role in determination of the vascularity observed in *VHL*-associated tumours and the formation of disease-associated cysts that may result from enhanced vascular permeability. Four differentially spliced VEGF isoforms have been described, the larger two of which are cell associated ($VEGF_{189}$ and $VEGF_{206}$), while the smaller two are secreted proteins ($VEGF_{121}$ and $VEGF_{165}$) (Ferrara et al, 1991; Breier et al, 1992). In PTECs, we observed increases in $VEGF_{121\ and\ 165}$ mRNA levels peaking at 2–4 h after exposure to HGF/SF, while expression of the cell-associated ($VEGF_{189\ and\ 206}$) isoforms was not detected. Enhanced VEGF mRNA levels may be induced by a number of events, including growth factor stimulation, induction

© Cancer Research Campaign 1998

of hypoxia and oncogene activation (Finkenzeller et al, 1995; Grugel et al, 1995; Ikeda et al, 1995; Rak et al, 1995). In addition, Silvagno et al (1995) have reported that HGF/SF induces expression of angiogenic factors, including VEGF in vivo, and that a neutralizing antibody to VEGF partly prevented HGF-induced angiogenesis. Our results are consistent with these observations and the concept that HGF/SF has a role in the control of renal angiogenesis during development and that this role is, at least in part, mediated through the induction of VEGF expression. As peak elongin mRNA levels occur up to 6 h after the observed VEGF peak, it seems unlikely that changes in VEGF expression result from enhanced transcriptional elongation. Up-regulation of VEGF expression in response to growth factors, such as platelet-derived growth factor, epidermal growth factor and tumour-promoting agent (phorbol ester), results from promoter activation (Grugel et al, 1995; Rak et al, 1995), whereas the VEGF response to hypoxia appears to be primarily mediated at the mRNA stabilization level (Finkenzeller et al, 1995; Ikeda et al, 1995). The *pVHL*-induced changes in VEGF expression that have been demonstrated in vitro appear to be mediated post-transcriptionally through mRNA stabilization and not by influencing transcriptional elongation (Gnarra et al, 1996; Iliopoulos et al, 1996).

Finally, our data support a role for the HGF/SF receptor (the c-*met* proto-oncogene) as a delayed early-response gene in PTECs (peak levels are reached at 8 h after stimulation). Similar results have previously been described by Boccaccio et al (1994) in an epithelial lung adenocarcinoma cell line, showing a peak in c-*met* mRNA expression at 4 h, which had fallen to basal levels by 16 h, after stimulation with HGF/SF. Thus, a feedback loop may exist whereby exposure of PTECs to HGF/SF results in the induction of c-*met* receptor expression, however it is presently unclear whether c-*met* expression is specifically induced by HGF/SF, or whether this represents a more general mitogenic response.

In summary, we have confirmed and extended previous studies that have demonstrated an association between HGF/SF-induced renal tubular mitogenesis and increased expression of c-*myc*, c-*fos*, c-*met* and VEGF. In addition, we have demonstrated that HGF/SF increases elongin mRNA levels. No profound changes in VHL mRNA levels were observed after HGF/SF, and current evidence suggests that alterations in the VHL/elongin pathway function associated with modulation of transcriptional elongation are unlikely to be an important mediator of HGF/SF-induced renal cell mitogenesis.

### ACKNOWLEDGEMENTS

This work was funded by the Cancer Research Campaign (CRC) and the Hope Hospital Renal Endowment fund. We are grateful to Dr E Gherardi for the supply of HGF/SF and critical reading of the manuscript, Mr RWG Johnson for the provision of clinical material, Dr PEC Brenchley for advice on renal tubular growth condtions and Dr A Darvill for technical assistance.

### REFERENCES

Aso T, Lane WS, Conaway JW and Conaway RC (1995) Elongin (SIII) – a multisubunit regulator of elongation by RNA polymerase II. *Science* **269**: 1439–1443

Boccaccio C, Gaudino G, Gambarotta G, Galimi F and Comoglio PM (1994) Hepatocyte growth factor (HGF) receptor expression is inducible and is part of the delayed early response to HGF. *J Biol Chem* **269**: 12846–12851

Breier G, Albrecht U, Sterrer S and Risau W (1992) Expression of vascular endothelial growth factor during embryonic angiogenesis and endothelial cell differentiation. *Development* **114**: 521–532

Brown LF, Berse B, Jackman RW, Tognazzi K, Manseau EJ, Dvorak HF and Senger DR (1993) Increased expression of vascular permeability factor (vascular endothelial growth factor) and its receptors in kidney and bladder carcinomas. *Am J Pathol* **143**: 1255–1262

Cantley LG, Barros EJG, Gandhi M, Rauchman M and Nigam SK (1994) Regulation of mitogenesis, motogenesis and tubulogenesis by hepatocyte growth-factor in renal collecting duct cells. *Am J Physiol* **267**: F271–F280

Chang HG, Okuda T, Nomura Y, Nagao T, Nakamura T, Kurokawa K and Katoh T (1996) Serum hepatocyte growth-factor concentration in patients with various degrees of chronic-renal-failure. *Nephrology* **2**: 175–179

Chen F, Kishida T, Duh F-M, Renbaum P, Orcutt ML, Schmidt L and Zbar B (1995) Suppression of growth of renal carcinoma cells by the von Hippel–Lindau tumor suppressor gene. *Cancer Res* **55**: 4804–4807

Clifford SC, Thomas DJ, Neal DE and Lunec J (1994) Increased MDR1 gene transcript levels in high grade carcinoma of the bladder determined by quantitative PCR-based assay. *Br J Cancer* **69**: 680–686

Connolly DT, Heuvelman DM, Nelson R, Olander JV, Eppley BL, Elfino JJ, Siegel NR, Leimgruber RM and Feder J (1989) Tumor vascular-permeability factor stimulates endothelial-cell growth and angiogenesis. *J Clin Invest* **84**: 1470–1478

Detrisac CJ, Sens MA, Garvin AJ, Spicer SS and Sens DA (1984) Tissue-culture of human kidney epithelial cells of proximal tubule origin. *Kidney Int* **25**: 383–390

Dressler GR and Douglass EC (1992) Pax-2 is a DNA binding protein expressed in embryonic kidney and Wilms' tumour. *Proc Natl Acad Sci USA* **89**: 1179–1183

Duan DR, Pause A, Burgess WH, Aso T, Chen DYT, Garret KP, Conaway RC, Conaway JW, Linehan WM and Klausner RD (1995) Inhibition of transcription elongation by the VHL tumour suppressor protein. *Science* **269**: 1402–1407

Eick D, Polack A, Kofler E, Lenoir GM, Rickinson AB and Bornkamm GW (1990) Expression of P0 and P3-RNA from the normal and translocated c-*myc* allele in Burkitt's lymphoma cells. *Oncogene* **9**: 1397–1402

Ferrara N, Houck KA, Jakeman LB, Winer J and Leung DW (1991) The vascular endothelial growth-factor family of polypeptides. *J Cell Biochem* **47**: 211–218

Finkenzeller G, Technau A and Marme D (1995) Hypoxia-induced transcription of the vascular endothelial growth-factor gene is independent of functional ap-1 transcription factor. *Biochem Biophys Res Commun* **208**: 432–439

Fleischmann J and Huntley NH (1994) Renal tumours. In *Clinical Urology*, Krane RJ, Siroky MB and Fitzpatrick JM. (eds), pp. 359–373. JB Lipincott: Philadelphia

Foster K, Prowse A, van den Berg A, Fleming S, Hulsbeek MMF, Crossey PA, Richards FM, Cairns P, Affara NA, Ferguson-Smith MA, Buys CHCM and Maher ER (1994) Somatic mutations of the von Hippel–Lindau disease tumour suppressor gene in nonfamilial clear cell renal carcinoma. *Hum Mol Genet* **3**: 2169–2173

Gnarra JR and Dressler GR (1995) Expression of *pax*-2 in human renal cell carcinoma and growth inhibition by antisense oligonucleotides. *Cancer Res* **55**: 4092–4098

Gnarra JR, Tory K, Weng Y, Schmidt L, Wei MH, Li H, Latif F, Liu S, Chen F, Duh FM, Lubensky I, Duan DR, Florence C, Pozatti R, Walther MM, Bander NH, Grossman HB, Brauch H, Pomer S, Brooks JD, Isaacs WB, Lerman MI, Zbar B and Linehan WM (1994) Mutations of the VHL tumor suppressor gene in renal carcinoma. *Nature Genet* **7**: 85–90

Gnarra JR, Zhou SB, Merrill MJ, Wagner JR, Krumm A, Papavassiliou E, Oldfield EH, Klausner RD and Linehan WM (1996) Posttranscriptional regulation of vascular endothelial growth-factor messenger-RNA by the product of the VHL tumor-suppressor gene. *Proc Natl Acad Sci USA* **93**: 10589–10594

Grugel S, Finkenzeller G, Weindel K, Barleon B and Marme D (1995) Both v-Ha-Ras and v-raf stimulate expression of the vascular endothelial growth-factor in NIH 3T3 cells. *J Biol Chem* **270**: 25915–25919

Harris RC, Burns KD, Alattar M, Homma T and Nakamura T (1993) Hepatocyte growth-factor stimulates phosphoinositide hydrolysis and mitogenesis in cultured renal epithelial-cells. *Life Sci* **52**: 1091–1100

Herman JG, Latif F, Weng Y, Lerman MI, Zbar B, Liu S, Samid D, Duan DR, Gnarra JR, Linehan WM and Baylin SB (1994) Silencing of the VHL tumor suppressor gene by DNA methylation in renal carcinoma. *Proc Natl Acad Sci USA* **91**: 9700–9704

Horikoshi T, Danenberg KD, Stadlbauer THW, Vlkenandt M, Shea LCC, Aigner K, Gustavsson B, Leichman L, Frosing R, Ray M, Gibson NW, Spears CP and Danenberg PV (1992) Quantitation of thymidylate synthase dihydrofolate reductase and DT-diaphorase gene expression in human tumors using the polymerase chain reaction. *Cancer Res* **52**: 108–116

© Cancer Research Campaign 1998 

Ikeda E, Achen MG, Brier G and Risau W (1995) Hypoxia-induced transcriptional activation and increased messenger-RNA stability of vascular endothelial growth-factor in C6 glioma-cells. *J Biol Chem* **270**: 19761–19766

Iliopoulos O, Kibel A, Gray S and Kaelin Jr WG (1995) Tumour suppression by the human von Hippel–Lindau gene product. *Nature Med* **1**: 822–826

Iliopoulos O, Levy AP, Jiang C, Kaelin WG and Goldberg MA (1996) Negative regulation of hypoxia-inducible genes by the von Hippel–Lindau protein. *Proc Natl Acad Sci USA* **93**: 10595–10599

Johnson M, Kochhar K, Nakamura T and Iyer A (1995) Hepatocyte growth factor-induced signal transduction in two normal mouse epithelial cell lines. *Biochem Mol Biol Int* **36**: 465–474

Kawaida K, Matsumoto K, Shimazu H and Nakamura T (1994) Hepatocyte growth-factor prevents acute renal failure and accelerates renal regeneration in mice. *Proc Natl Acad Sci USA* **91**: 4357–4361

Keck PJ, Hauser SD, Krivi G, Sanzo K, Warren T, Feder J and Connolly DT (1989) Vascular-permeability factor: an endothelial-cell mitogen related to PDGF. *Science* **246**: 1309–1312

Kibel A, Iliopoulos O, DeCaprio JA and Kaelin WG (1995) Binding of the von Hippel–Lindau tumor suppressor protein to Elongin B and C. *Science* **269**: 1444–1446

Kohlhuber F, Strobl LJ and Eick D (1993) Early down-regulation of c-*myc* in dimethylsulfoxide-induced mouse erythroleukemia (mel) cells is mediated at the P1/P2 promoters. *Oncogene* **8**: 1099–1102

Krumm A, Meulia T, Brunvand M and Groudine M (1992) The block to transcriptional elongation within the human c-*myc* gene is determined in the promoter–proximal region. *Genes Dev* **6**: 2201–2213

Kuzmin I, Duh F-M, Latif F, Geil L, Zbar B and Lerman MI (1995) Identification of the promoter of the human von Hippel–Lindau disease tumor suppressor gene. *Oncogene* **10**: 2185–2194

Latif F, Tory K, Gnarra J, Yao M, Duh F-M, Orcutt ML, Stackhouse T, Kuzmin I, Mosi W, Geil L, Schmidt L, Zhou F, Li H, Wei MH, Chen F, Glenn G, Choyke P, Walther MM, Weng Y, Duan DR, Dean M, Glavac D, Richards FM, Crossey PA, Ferguson-Smith MA, Le Paslier D, Chumakov I, Cohen D, Chinault AC, Maher ER, Linehan WM, Zbar B and Lerman MI (1993) Identification of the von Hippel–Lindau disease tumour suppressor gene. *Science* **260**: 1317–1320

Lee S, Chen DYT, Humphrey JS, Gnarra JR, Linehan WM and Klausner RD (1996) Nuclear-cytoplasmic localization of the von Hippel–Lindau tumor suppressor gene product is determined by cell-density. *Proc Natl Acad Sci USA* **93**: 1770–1775

Leung DW, Cachianes G, Kuang WJ, Goeddel DV and Ferrara N (1989) Vascular endothelial growth-factor is a secreted angiogenic. *Science* **246**: 1306–1309

Maher ER, Yates JRW, Harries R, Benjamin C, Harris R and Ferguson-Smith MA (1990) Clinical features and natural history of von Hippel–Lindau disease. *Q J Med* **77**: 1151–1163

Pause A, Lee S, Worrell RA, Chen DYT, Burgess WH, Linehan WM and Klausner RD (1997) The von Hippel–Lindau tumor-suppressor gene product forms a stable complex with human CUL-2 a member of the Cdc53 family of proteins. *Proc Natl Acad Sci USA* **94**: 2156–2161

Rak J, Mitsuhashi Y, Bayko L, Filmus J, Shirasawa S, Sasazuki T and Kerbel RS (1995) Mutant ras oncogenes up-regulate VEGF/VPF expression – implications for induction and inhibition of tumor angiogenesis. *Cancer Res* **55**: 4575–4580

Richards FM, Schofield PN, Fleming S and Maher ER (1996) Expression of the von Hippel–Lindau disease tumour suppressor gene during human embryogenesis. *Hum Mol Genet* **5**: 639–644

Schmidt L, Duh FM, Chen F, Kishida T, Glenn G, Choyke P, Scherer SW, Zhuang Z, Lubensky I, Dean M, Allikmets R, Chidambaram A, Bergerheim UR, Feltis JT, Casadevall C, Zamarron A, Richard S, Lips CJM, McCellan MW, Lap-Chee T, Geil L, Orcutt ML, Stackhouse T, Lipan J, Slife L, Brauch H, Decker J, Niehans G, Hughson MD, Moch H, Lerman MI, Linehan WM and Zbar B (1997) Germline and somatic mutations in the tyrosine kinase domain of the MET proto-oncogene in papillary renal carcinomas. *Nature Genet* **16**: 68–73

Shuin T, Kondo K, Torigoe S, Kishida T, Kubota Y, Hosaka M, Nagashima Y, Kitamura H, Latif F, Zbar B, Lerman MI and Yao M (1994) Frequent somatic mutations and loss of heterozygosity of the von Hippel–Lindau tumor suppressor gene in primary human renal cell carcinomas. *Cancer Res* **54**: 2852–2855

Siemeister G, Weindel K, Mohrs K, Barleton B, Martiny-Baron G and Marme D (1996) Reversion of deregulated expression of vascular endothelial growth factor in human renal carcinoma cells by von Hippel–Lindau tumour suppressor protein. *Cancer Res* **56**: 2299–2301

Silvagno F, Follenzi A, Arese M, Prat M, Giraudo E, Gaudino G, Camussi G, Comoglio PM and Bussolino F (1995) *In-vivo* activation of *met* tyrosine kinase by heterodimeric hepatocyte growth-factor molecule promotes angiogenesis. *Arter Thromb Vasc Biol* **15**: 1857–1865

Spencer CA and Groudine M (1991) Control of c-myc regulation in normal and neoplastic cells. *Adv Cancer Res* **56**: 1–48

Stackhouse TM, Kishida T, Chase D, Ferris DK, Kuzmin I, Geil L, Orcutt ML, Sakashita N, Takeya M, Renbaum P and Zbar B (1996) Phosphorylation of the von Hippel–Lindau disease tumour suppressor protein by casein kinase II. (in press)

Strobl LJ and Eick D (1992) Hold back of RNA polymerase-II at the transcription start site mediates down-regulation of c-*myc in vivo*. *EMBO J* **11**: 3307–3331

Wizigmann-Voos S, Breier G, Risau W and Plate KH (1995) Up-regulation of vascular endothelial growth factor and its receptor in von Hippel–Lindau disease-associated and sporadic hemangioblastomas. *Cancer Res* **55**: 1358–1364

Woolf AS, Kolatsijoannou M, Hardman P, Andermarcher E, Moorby C, Fine LG, Jat PS, Noble MD and Gherardi E (1995) Roles of hepatocyte growth factor/scatter factor and the met receptor in the early development of the metanephros. *J Cell Biol* **128**: 171–184

Yao M, Shuin T, Misaki H and Kubota Y (1988) Enhanced expression of c-myc and epidermal growth factor receptor (C-erbB-1) genes in primary human renal cancer. *Cancer Res* **48**: 6753–6757

 © Cancer Research Campaign 1998